# Intimate partner violence against women during pregnancy: a systematic review and meta-analysis protocol for producing global and regional estimates

**DOI:** 10.1186/s13643-023-02232-2

**Published:** 2023-06-30

**Authors:** Leah Schrubbe, Claudia García-Moreno, Lynnmarie Sardinha, Heidi Stöckl

**Affiliations:** 1 Gender Violence and Health Centre, Global Health and Development, London School of Hygiene & Tropical Medicine (LSHTM), London, UK; 2 Department of Sexual and Reproductive Health and Research, The UNDP-UNFPA-UNICEF-WHO-World Bank Special Programme of Research, Development and Research Training in Human Reproduction (HRP), World Health Organization, Geneva, Switzerland; 3 Institute for Medical Information Processing, Biometry and Epidemiology, Faculty of Medicine, LMU Munich, Munich, Germany

**Keywords:** Intimate partner violence, Violence against women, Domestic violence, Pregnancy, Systematic review, Global estimates, Prevalence, Antenatal/postnatal

## Abstract

**Background:**

Intimate partner violence is a devastating human rights violation and public health problem with high prevalence rates globally. Intimate partner violence during pregnancy is associated with devastating maternal, perinatal, and neonatal health effects. We present the protocol for a systematic review and meta-analysis to estimate the global lifetime prevalence of intimate partner violence during pregnancy.

**Methods:**

This review aims to systematically synthesize the evidence on the global prevalence of violence against women by intimate partners during pregnancy using available population-based data. A comprehensive search of MEDLINE, EMBASE, Global Health, PsychInfo, and Web of Science databases will be conducted to identify all relevant articles. Manual searches will be conducted in Demographic and Health Survey (DHS) data reports and websites of national statistics and/or other offices. DHS data analysis will also be conducted. Based on inclusion and exclusion criteria, titles and abstracts will be screened for eligibility. Then, full-text articles will be assessed for eligibility. The following data will be extracted from included articles: study characteristics, population characteristics (e.g., ever-partnered, currently partnered, or any women, and age range), violence characteristics (e.g., type of violence, and perpetrator), estimate type (e.g., intimate partner violence during any pregnancy or during last pregnancy), subpopulation type (e.g., by age, marital status, urban/rural), prevalence estimate, and key quality indicators. A hierarchical Bayesian meta-regression framework will be used. This multilevel modelling approach will use survey-specific, country-specific, and region-specific random effects to pool observations. This modelling technique will be used to estimate global and regional prevalence.

**Discussion:**

This systematic review and meta-analysis will provide estimates on the global and regional prevalence of intimate partner violence during pregnancy and contribute to monitoring progress towards Sustainable Development Goal (SDG) Target 5.2 on eliminating violence against women and to SDG Targets 3.1 and 3.2 on reducing maternal mortality and neonatal mortality.

Given the significant health impacts of intimate partner violence during pregnancy, potential for intervention, and urgency to address violence and improve health, this review will provide critical evidence to governments, non-governmental organizations, and policymakers on the magnitude of violence during pregnancy. It will also inform effective policies and programs to prevent and respond to intimate partner violence during pregnancy.

**Systematic review registration:**

PROSPERO ID CRD42022332592.

**Supplementary Information:**

The online version contains supplementary material available at 10.1186/s13643-023-02232-2.

## Background

Intimate partner violence, defined as acts or threats of physical, sexual, or emotional harm by current or former partners, is a devastating human rights violation and public health concern [1]. It is highly prevalent around the world, and one of the most common forms of violence against women globally [1]. It is estimated that over 1 in 4 ever-partnered women aged 15 years and older will experience physical and/or sexual intimate partner violence in their lifetime [2]. Among 19 countries based on data from Demographic and Health Surveys (DHS) and International Violence Against Women Surveys data [3], existing prevalence estimates of intimate partner violence during pregnancy range from 2 to 13.5%, and from 0.7 to 55.1% among 126 cross-sectional and cohort studies (mostly clinic- or hospital-based) from 52 countries worldwide [4]. Intimate partner violence can have devastating effects on mothers, foetuses, and infants; global evidence suggests women who are subjected to intimate partner violence during pregnancy are more likely to experience pregnancy termination, and are at increased risk for miscarriages, low birth weight babies and preterm birth [5]. For example, a population-based study in Tanzania found that even after adjusting for other explanatory factors, women who experienced intimate partner violence were 1.6 (95% CI 1.06–1.60) times more likely to report a pregnancy loss and 1.9 (95% CI 1.30–2.89) times more likely to report an induced abortion [6]. Intimate partner violence in pregnancy is also associated with maternal depression and other perinatal mental health problems [5]. A longitudinal study from Recife in Brazil established that women reporting the highest frequency of psychological violence were more likely to have postnatal depression even after adjustment (adjusted OR 2.29, 95% CI 1.15–4.57), and women who reported physical or sexual violence in pregnancy being 3.28 times as likely to report postnatal depression (OR 3.28, 2.29–4.70) [7]. Postnatal depression and other perinatal mental health problems in turn are associated with infant and child health and development problems. Importantly, pregnancy is a time when women are most likely to be in touch with health services, including for antenatal and postnatal care, which offers opportunities for women to disclose and for healthcare providers to identify intimate partner violence and to provide the relevant care, support, and referrals as needed [3, 8].

Growing recognition of the high prevalence and significant health and other impacts of intimate partner violence have led to Sustainable Development Goal (SDG) Target 5.2 on the elimination of all forms of violence against women and girls [9]. Quality evidence on the global prevalence of intimate partner violence during pregnancy is needed and will contribute to addressing this problem and to monitoring progress towards this SDG. This manuscript presents the protocol for a systematic review and meta-analysis to estimate the global lifetime prevalence of intimate partner violence during pregnancy from available population-based data.

## Methods/design

This review builds on methodology used for previous systematic reviews on the global prevalence of intimate partner violence, albeit not specific to pregnancy [2, 10–13]. It will include population-based studies/surveys that are nationally and/or sub-nationally representative identified by an updated systematic review. This includes the Demographic and Health Surveys (DHS), the World Health Organization (WHO) multi-country study on women’s health and domestic violence, and other national surveys/studies conducted by national statistics offices or others. The existing WHO Database on Prevalence of Violence against Women and data extraction form on global prevalence of intimate partner violence will be adapted and expanded to include intimate partner violence during pregnancy. The review excludes studies that were solely based on hospital-based data or related service data or that relied on recruitment through lay health care workers, given that not all women might give birth or register their pregnancy at a hospital or with a lay health care worker. Given their sampling framework, these data from hospital-based settings result in prevalences that are not representative or generalizable to the wider population of pregnant women [14]. The results of this review will be reported according to the Preferred Reporting Items for Systematic Review and Meta-Analysis (PRISMA) guidelines. For this protocol, the PRISMA statement for Protocols (PRISMA-P) was used for reporting (see Additional file 1). This review was registered in the International Prospective Register of Systematic Reviews (PROSPERO) (registration ID: CRD42022332592).

### Aims of the review and research questions

This review aims to systematically summarize the evidence on the global prevalence of intimate partner violence during pregnancy among women using population-based data.

The specific review questions are the following:

1. What is the lifetime prevalence of intimate partner violence (physical; sexual; physical and/or sexual combined; psychological; physical, sexual, and/or psychological combined) during pregnancy in women?
2. What is the prevalence of ever- and/or currently pregnant women who were beaten in their most recent pregnancy by the father of their child?
3. How does prevalence of intimate partner violence during pregnancy vary by age group, rural and urban setting, marital status, number of living children, education, wealth, and also across global regions?
4. What is the lifetime prevalence of women who were kicked or punched in the abdomen while pregnant?
5. What is the prevalence of women who were beaten by the father of the child they were carrying during their last pregnancy?
6. What is the prevalence of women who were beaten during their last pregnancy by the same person who beat them prior to the pregnancy?
7. What is the prevalence of women for whom the abuse during pregnancy was preceded by intimate partner violence before the pregnancy? What is the prevalence of women for whom the violence started with the pregnancy?
8. What is the prevalence of women who were beaten during pregnancy by their partner for whom the beating worsened, stayed the same, or lessened in frequency or severity compared to before the pregnancy?

### Inclusion criteria

- Location: any country
- Study design: population-based cross-sectional survey or cohort study
- Population: ever-pregnant or currently pregnant women aged 15 years or older
- Results include prevalence of male intimate partner violence during pregnancy (physical, sexual, and/or psychological).
- Use acts-based measures of intimate partner violence—meaning that women are asked whether they experienced a specific act of violence, such as being beaten or hit, compared to a broader question such as whether they experienced violence or were a victim of violence.

### Exclusion criteria

- Duplicate reports and/or publications of the same data. The less comprehensive/complete and up-to-date version will be excluded. Studies of different years will be captured.
- Inappropriate study design (case report, case series, systematic review, abstract only, editorial, conference, books, qualitative, ecological, randomized controlled trial, case–control, administrative data such as health statistics or police statistics on reported crimes)
- Estimates are not population-based
- Minimum reporting criteria (prevalence) are not available
- No full text available

### Databases and search strategy

A comprehensive search of MEDLINE, EMBASE, Global Health, PsychInfo, and Web of Science databases will be conducted upon publication of the protocol in Prospero to identify all relevant articles regardless of the language of publication. We plan to regularly update the search alongside the updating of the global estimates on violence against women and girls [11]. Since this review builds on previous searches/reviews [2, 10–13] of population-based estimates of intimate partner violence prevalence from studies between 2000 and 2018, in the WHO Global Database of VAW Prevalence, a new search with a search term for pregnancy will be conducted to identify any additional data published since 31 December 2018. Included studies from previous searches/reviews will be rescreened for data on intimate partner violence during pregnancy. The search strategy is shown in Additional file 2.

Manual searches will be conducted of DHS data reports and websites of national statistical offices. DHS microdata analysis will also be conducted by calculating the prevalence of violence during pregnancy being committed by the former or current partner or boyfriend of the woman, as this is not explicitly stated in the DHS reports. Data will be excluded if it is administrative data such as health statistics or police statistics on reported crimes, as these only represent a subset of women and are not population-based data.

### Study selection

Systematic review screening software (Rayyan) will be used by two independent reviewers to screen titles and abstracts for eligibility. Then, full-text articles deemed potentially eligible will be retrieved and independently assessed for eligibility. Any eligibility disagreements will be discussed with a third reviewer to reach consensus, if needed. Reference management software (ENDNOTE X9) will be used to manage articles meeting inclusion criteria.

### Data extraction

A reviewer will extract data using a data extraction form (excel spreadsheet). A second reviewer will do a random quality control check on at least 10% of the data. After screening by both reviewers, they should achieve a kappa score of 0.80 or greater. If they achieve less than 80%, both reviewers will double extract all data. Any disagreements will be discussed with a third extractor. Studies in languages other than English will be screened and extracted by a native speaker. Google Translate will be used for preliminary screening of the foreign language abstract only to assess if the study is based on empirical data. If not, it will be excluded. Once this is confirmed, we will engage a native speaker or someone with good language skills in the non-English language to screen the abstract and extract the data if applicable. Extracted data will be checked through cross tabulations and range checks and checked for implausible values before data analysis. Extracted data from each study will include:

1. Study characteristics: study title, author, publication year, country, year(s) of data collection, setting (urban/rural/mixed), survey type (DHS, WHO, or other population-based survey)
2. Population characteristics: denominator information (ever-pregnant, currently pregnant, ever-partnered, currently partnered, or any women), age range of sample
3. Violence characteristics: type of violence (physical intimate partner violence only, sexual intimate partner violence only, psychological intimate partner violence only, physical and/or sexual intimate partner violence combined, or physical, sexual, and/or psychological intimate partner violence combined), time frame (during any pregnancy or during last pregnancy), perpetrator of violence (anyone [including husband/partner], any husband/partner, current husband/partner, or former husband/partner)
4. Estimate type: prevalence estimate type (intimate partner violence during any pregnancy, intimate partner violence during last pregnancy, women reporting being punched or kicked in the abdomen while pregnant, beating started during pregnancy, woman was beaten in most recent pregnancy by father of child, woman lives with person who beat her while pregnant, woman reports that the same person had beaten her before pregnancy, woman reported that the beating became worse, stayed the same, or became less than before pregnancy). This information differs from the previous review on the global prevalence of intimate partner violence and non-partner sexual violence and captures pregnancy-specific circumstances of violence during pregnancy.
5. Subpopulation type: within country, subpopulation of the estimate (by age, marital status, urban/rural, number of living children, education, or wealth quintile)
6. Estimate: prevalence point estimate, type of confidence interval or uncertainty interval, lower and upper intervals, standard error, numerator, denominator
7. Key quality indicators: study specified specific interviewer training on administering questions on violence against women, specific violence against women survey or a module in a broader survey

### Quality assessment

The Joanna Briggs Institute (JBI) critical appraisal checklist for prevalence studies will be used to assess the methodological quality of studies [15]. In addition, quality of included studies will be assessed on whether they were dedicated or violence against women-specific, meaning that the study was designed to investigate violence against women or girls compared to a module of questions on intimate partner violence being part of a broader study on another topic. This is important as violence against women specific surveys are more likely to train interviewers on the sensitive content and design the study and questionnaire in a way that maximizes confidentiality and rapport to reduce underreporting of experiences of violence.

### Data analysis

Data from this systematic review will be used to answer the identified research questions. An overall descriptive table of included studies will be built using data from each study. For the meta-analysis, a hierarchical Bayesian meta-regression framework will be used. This multilevel modelling approach will use survey-specific, country-specific, and region-specific random effects to pool observations and improve accuracy of estimates. This model structure is based on similar meta-regressions of global estimates of health indicators [10, 16–24]. Regions will be defined based on the Global Burden of Diseases classification [25]. The modelling approach will consider heterogeneous age groups (using an age-standardising approach), country-specific age and time trends (using splines) and adjust for key survey differences, for example, type of violence, all women versus ever-partnered women; ever-partnered versus currently partnered women, ever-pregnant versus currently pregnant women, time frame of violence during any pregnancy versus during last pregnancy, urban or rural versus national, perpetrator of violence (spouse only or any intimate partner) and quality ratings through covariate modelling. This modelling technique will be used to estimate the global and regional prevalence along with 95% uncertainty intervals. Forest plots and tables will be used to present results and funnel plots will be visualized for symmetry (publication bias) if we have 10 or more studies in the meta-analysis. The performance of models will be assessed using posterior predictive checks, and both in-sample and out-of-sample comparisons. Graphical posterior predictive checks will enable visual assessment of how well simulations from the fitted model compare to the observed data [26].

## Discussion

This systematic review and random-effects meta-analysis will provide estimates on the global prevalence of intimate partner violence during pregnancy using population-based data. It will help establish the magnitude and nature of intimate partner violence during pregnancy and thereby contribute to monitoring progress towards SDG Target 5.2 on eliminating violence against women. Given the significant health impacts intimate partner violence during pregnancy can have on maternal, perinatal, and neonatal health, this review will also contribute important evidence relevant to SDG Targets 3.1 and 3.2 on reducing maternal mortality and neonatal mortality.

Over the past decade, governments have made commitments towards addressing violence against women, including increased research and investment in population-based surveys on violence against women. These data and estimates will contribute to the robust and expanding evidence base and will support the inclusion of intimate partner violence in maternal health programs and tracking of progress over time. The findings of this review will help inform governments, non-governmental organizations, and policymakers of the magnitude of the problem and guide development of effective policies and programs to prevent and respond to intimate partner violence during pregnancy. While violence also occurs in same sex couples, the majority of population-based surveys on violence against women focus on violence by men against women. This review may be updated in the future to monitor progress made towards addressing intimate partner violence during pregnancy. In addition to providing prevalence estimates, this review will allow for identification of challenges and ways to improve measures/instruments and reporting of data on intimate partner violence during pregnancy.

Violence during pregnancy presents additional health risks that do not apply to other stages in women’s lives. During pregnancy, intimate partner violence can result in maternal health effects ranging from insufficient or inconsistent antenatal care and inadequate weight gain to maternal depression and other mental health problems and to maternal mortality. Intimate partner violence during pregnancy can also result in obstetric and gynecological problems including miscarriage and neonatal health effects ranging from low birth weight and preterm birth to neonatal death. In addition, pregnancy may be a time of increased vulnerability for intimate partner violence due to the physical, social, and economic demands of this period, but it also offers increased opportunity for intervention. The increased and repeated interactions with the healthcare system through antenatal and postnatal care represent a window of opportunity to address intimate partner violence. In the long term, identifying and addressing violence during pregnancy and its accompanying impacts on mental health also contributes to improved child health and development outcomes and reduces the likelihood of so-called intergenerational transmission of violence, thereby contributing to prevention of violence in later life [27]. Given the significant health impacts of intimate partner violence during pregnancy, potential for intervention, and urgency to address the SDGs, this review will provide critical evidence towards the global burden of this human rights and public health issue.

### Supplementary Information

**Additional file 1.** PRISMA-P 2015 Checklist.

**Additional file 2.** Search strategy.

## Data Availability

Not applicable.

## Abbreviations

DHS: Demographic and Health Survey
JBI: Joanna Briggs Institute
PRISMA: Preferred Reporting Items for Systematic Review and Meta-Analysis
PRISMA-P: Preferred Reporting Items for Systematic Review and Meta-Analysis Protocol
PROSPERO: International Prospective Register of Systematic Reviews
SDG: Sustainable development goal
WHO: World Health Organization
