# Correction: Intimate partner violence against women during pregnancy: a systematic review and meta‑analysis protocol for producing global and regional estimates

**DOI:** 10.1186/s13643-023-02297-z

**Published:** 2023-08-04

**Authors:** Leah Schrubbe, Claudia García‑Moreno, Lynnmarie Sardinha, Heidi Stöckl

**Affiliations:** 1 Gender Violence and Health Centre, Global Health and Development, London School of Hygiene & Tropical Medicine (LSHTM), London, UK; 2 Department of Sexual and Reproductive Health and Research, The UNDP‑UNFPA‑UNICEF‑WHO‑World Bank Special Programme of Research, Development and Research Training in Human Reproduction (HRP), World Health Organization, Geneva, Switzerland; 3 Institute for Medical Information Processing, Biometry and Epidemiology, Faculty of Medicine, LMU Munich, Munich, Germany

**Correction: Syst Rev 12, 107 (2023)**

10.1186/s13643-023-02232-2

Following publication of the original article [1], an error was identified under the Open Acess license statement, it should be changed to the CC BY 3.0 IGO version as shown in this article. The original article has been corrected.
