# Supplementary material for: Intimate partner violence against women during pregnancy: a systematic review and meta-analysis protocol for producing global and regional estimates
Source: Syst Rev. 2023 Jun 30;12:107. doi: 10.1186/s13643-023-02232-2 (PMC10311898; doi:10.1186/s13643-023-02232-2)
Supplement: Supplementary file 2 — Additional file 2. Search strategy. [file 13643_2023_2232_MOESM2_ESM.docx]

**Search Strategy**

| 1 | meta.ab. |
| --- | --- |
| 2 | synthesis.ab. |
| 3 | literature.ab. |
| 4 | published.ab. |
| 5 | extraction.ab. |
| 6 | search.ab. |
| 7 | medline.ab. |
| 8 | selection.ab. |
| 9 | sources.ab. |
| 10 | trials.ab. |
| 11 | review.ab. |
| 12 | articles.ab. |
| 13 | reviewed.ab. |
| 14 | english.ab. |
| 15 | language.ab. |
| 16 | randomized.hw. |
| 17 | trials.hw. |
| 18 | controlled.hw. |
| 19 | meta-analysis.pt. |
| 20 | review.pt. |
| 21 | or/1-20 |
| 22 | epidemiologic studies/ |
| 23 | exp case control studies/ |
| 24 | exp cohort studies/ |
| 25 | case control.tw. |
| 26 | (cohort adj (study or studies)).tw. |
| 27 | cohort analy$.tw. |
| 28 | (follow up adj (study or studies)).tw. |
| 29 | (observational adj (study or studies)).tw. |
| 30 | longitudinal.tw. |
| 31 | retrospective.tw. |
| 32 | cross sectional studies.tw. |
| 33 | cross sectional studies/ |
| 34 | or/22-33 |
| 35 | Animals/ |
| 36 | Humans/ |
| 37 | 35 not (35 and 36) |
| 38 | comment.pt. |
| 39 | letter.pt. |
| 40 | editorial.pt. |
| 41 | or/37-40 |
| 42 | domestic violence/ or partner violence/ or spouse abuse/ or spouse violence/ or domestic abuse/ or partner abuse.mp. [mp=title, abstract, heading word, table of contents, key concepts, original title, tests & measures] |
| 43 | *battered women/ |
| 44 | (intimate adj4 partner adj4 violence).tw. |
| 45 | (intimate adj4 partner adj4 abuse).tw. |
| 46 | (intimate adj4 partner adj4 victimi*).tw. |
| 47 | domestic abuse.tw. |
| 48 | spou$ abuse.tw. |
| 49 | dating violence.tw. |
| 50 | sexual abuse.tw. |
| 51 | ((partner or relationship or wom$n or domestic or spous*) adj4 (abus* or violen* or victimi* or batter*)).mp. |
| 52 | dating violence.tw. |
| 53 | sexual violence.tw. |
| 54 | rape.tw. |
| 55 | prevalence.tw. |
| 56 | cross‐sectional stud$.mp. [mp=title, abstract, heading word, table of contents, key concepts, original title, tests & measures] |
| 57 | survey.mp. [mp=title, abstract, heading word, table of contents, key concepts, original title, tests & measures] |
| 58 | health survey$.mp. [mp=title, abstract, heading word, table of contents, key concepts, original title, tests & measures] |
| 59 | pregnan*.mp. |
| 60 | or/42-54 |
| 61 | or/55-58 |
| 62 | 59 and 60 and 61 |
| 63 | 62 not 41 |
| 64 | 63 and (21 or 34) |
| 65 | limit 64 to yr="2019 - 2022" |
